# Fidaxomicin versus oral vancomycin for *Clostridioides difficile* infection among patients at high risk for recurrence based on real-world experience

**DOI:** 10.1017/ice.2024.145

**Published:** 2024-11

**Authors:** Natasha N. Pettit, Alison K. Lew, Cynthia T. Nguyen, Elizabeth Bell, Christopher J. Lehmann, Jennifer Pisano

**Affiliations:** 1 Department of Pharmacy, University of Chicago Medicine, Chicago, IL, USA; 2 Department of Medicine, Section of Infectious Diseases and Global Health, University of Chicago Medicine, Chicago, IL, USA

## Abstract

**Introduction:**

*Clostridioides difficile* infection (CDI) is a common nosocomial infection and is associated with a high healthcare burden due to high rates of recurrence. In 2021 the IDSA/SHEA guideline update recommended fidaxomicin (FDX) as first-line therapy. Our medical center updated our institutional guidelines to follow these recommendations, prioritizing FDX use among patients at high risk for recurrent CDI (rCDI).

**Methods:**

This pre- post- quasi-experimental study included patients with a presumptive diagnosis of CDI at risk for recurrence (age >/= 65 years, immunocompromised, severe CDI) that received vancomycin (VAN) or FDX between October 2019 to October 2022. Patients who received bezlotoxumab, had fulminant CDI, or received <10 days of the same antibiotic for their full treatment course were excluded. Patients were evaluated for rCDI within 8 weeks of completion of therapy, subsequent episodes of CDI within 12 months, and CDI-related admissions within 30 days.

**Results:**

Of 397 CDI regimens evaluated, 196 received VAN and 201 received FDX. Rates of rCDI (9.2% vs 10%, *P* = 0.86), subsequent CDI within 12 months of therapy completion of therapy (19.4% vs 26%, *P* = 0.12) and 30-day CDI-related readmissions (3% vs 4.5%, *P* = 0.6) were similar between patients who received VAN versus FDX.

**Conclusion:**

Outcomes were similar between patients treated with FDX and VAN for the treatment of CDI among those at high risk for rCDI, using our outlined criteria. Although we observed a trend toward lower rates of rCDI among immunocompromised patients, this finding was not significant. Further investigation is needed to determine which patients with CDI may benefit from FDX.

## Introduction

The Infectious Diseases Society of America (IDSA) and Society for Healthcare Epidemiology of America (SHEA) provided a focused update for the management of *Clostridioides difficile* infection (CDI) in June 2021, with fidaxomicin (FDX) recommended as first-line therapy for non-fulminant disease.^1^ FDX is recommended primarily based on clinical trial data showing a reduced rate of recurrence compared to oral vancomycin (VAN).^2–5^ FDX is thought to be associated with lower rates of recurrence due to its narrower spectrum of activity compared to VAN making it less disruptive to gut microbiota, its post-antibiotic effect, and bactericidal activity.

Although the IDSA/SHEA guidelines recommend a preference for FDX, high acquisition cost and limitations in coverage from payers remain a barrier to patient access and contribute to higher overall healthcare costs. The IDSA/SHEA guidelines note that VAN is a reasonable first-line option if resources are not available for obtaining FDX. Additionally, American College of Gastroenterology (ACG) guidelines for CDI management still recommend oral VAN as first-line therapy with the same level of evidence as FDX for non-fulminant disease for primary episodes.^6^

At our medical center, prior to the updated IDSA/SHEA guidelines, we routinely recommended VAN over FDX for non-fulminant CDI. Based on the updated guideline recommendations, we modified our recommendations to prefer FDX for patients with CDI meeting certain criteria. Given the high cost of FDX we chose to recommend FDX as first-line therapy only in patients at the greatest risk for recurrent CDI (rCDI). We sought to evaluate whether rates of rCDI at 8 weeks were lower among patients at high risk for rCDI that received FDX compared to VAN as the primary outcome.

## Methods

This single-center, pre-post quasi-experimental study included adult patients admitted to the University of Chicago Medicine between October 26, 2019 and October 25, 2022 with CDI at high risk for recurrence. We compared rates of rCDI (recurrent episode within 8 wk of index episode), subsequent episodes of CDI within 12 months, and CDI-related readmissions at 30 days between those who received FDX versus VAN. Patients were first identified by medication administration reports of oral VAN and FDX from the electronic health record (EHR) and then cross-referenced with laboratory diagnostics and rigorous manual chart review by an antimicrobial stewardship pharmacist to confirm CDI diagnosis. CDI was defined as a positive *Clostridioides difficile* test result and documentation of 3 or more loose stools over a 24-hour period. rCDI was defined as recurrent symptoms requiring retreatment with VAN or FDX within 8 weeks of the index episode. Repeat testing was not part of the definition. *Clostridioides difficile* testing was performed by either rectal swab (Roche 4800) or stool sample (Cepheid Genxpert® or BioFire Gastrointestinal FilmArray®) using nucleic acid amplification test (NAAT) for toxigenic genes. Enzyme immunoassay (EIA) testing for toxin A/B to confirm CDI diagnosis is not routinely performed at our institution. *C. difficile* rectal swabs are collected on all patients upon admission to our institution to assess for colonization, in accordance with an infection control protocol. If a patient with a positive screen develops symptoms consistent with CDI, they are initiated on therapy without the need for additional testing. Given the unconventional nature of this process, patients were considered to have a ‘presumptive’ diagnosis of CDI. Patients were considered high risk for rCDI if they met any of the following criteria: Age ≥65 years, immunocompromised status, and/or severe CDI defined as SCr ≥ 1.5 mg/dL and/or WBC >15,000 cells/mm^3^.

Immunocompromised status was defined as a history of solid organ transplantation or hematopoietic stem cell transplant; hematologic malignancy or solid tumor receiving chemotherapy within the past 3 months; concomitant use of immunosuppressive therapy defined as high dose steroid therapy (>0.5 mg/kg/day prednisone) or an equivalent immunosuppressive therapeutic regimen.

We updated our institutional CDI management pathway on October 1, 2021. Patients meeting the above criteria who received VAN between October 26, 2019 and October 25, 2020 and FDX between October 26, 2021 and October 25, 2022 were included. Patients were excluded for any of the following reasons: fulminant CDI, receipt of less than 10 days of CDI treatment, modification of CDI therapy during the treatment course, mortality within 30 days following the end of treatment, receipt of adjunctive therapy with bezlotoxumab. All patients initiated on VAN or FDX were reviewed by an antimicrobial stewardship program pharmacist as part of their daily workflow, as an electronic health record alert is triggered anytime an active order is entered for either VAN or FDX. Every patient started on CDI therapy was reviewed to ensure that they met criteria for CDI treatment and were initiated on appropriate regimens. Potential confounders that may have contributed to diarrhea or other gastrointestinal symptoms including recent laxative use within 48 hours of CDI treatment initiation, concomitant mycophenolate, concomitant tube feeds, recent chemotherapy, other infections known to be associated with diarrhea (eg, SARS-COV-2 infection, CMV colitis, *Helicobacter pylori* (*H. pylori*), enteropathogenic Escherichia coli (EPEC), concomitant antibiotic administration, and gastrointestinal comorbidities (eg, irritable bowel disease or inflammatory bowel disease (IBD), diverticulitis, pancreatic insufficiency) were additionally evaluated. No other changes to CDI management occurred during the study period. We also assessed rates of vancomycin-resistant enterococcus (VRE) and extended-spectrum beta-lactamase (ESBL) colonization following CDI treatment using infection control surveillance reports. Patients were deemed to have post-CDI treatment colonization with VRE or ESBL if the date of entry of colonization information into the medical record was after initiation of CDI treatment.

This project was formally determined to be quality improvement, not human subjects research, and was therefore not overseen by the Institutional Review Board, per institutional policy.

### Statistical analysis

Categorical data including baseline characteristics and outcomes were assessed using χ^2^ or Fisher’s exact testing where appropriate. Continuous data, including age, was evaluated using unpaired t-test. All statistical analyses were performed using GraphPad Prism version 10.0.0 for Windows, GraphPad Software, Boston, Massachusetts USA, www.graphpad.com.

## Results

**Figure 1.**
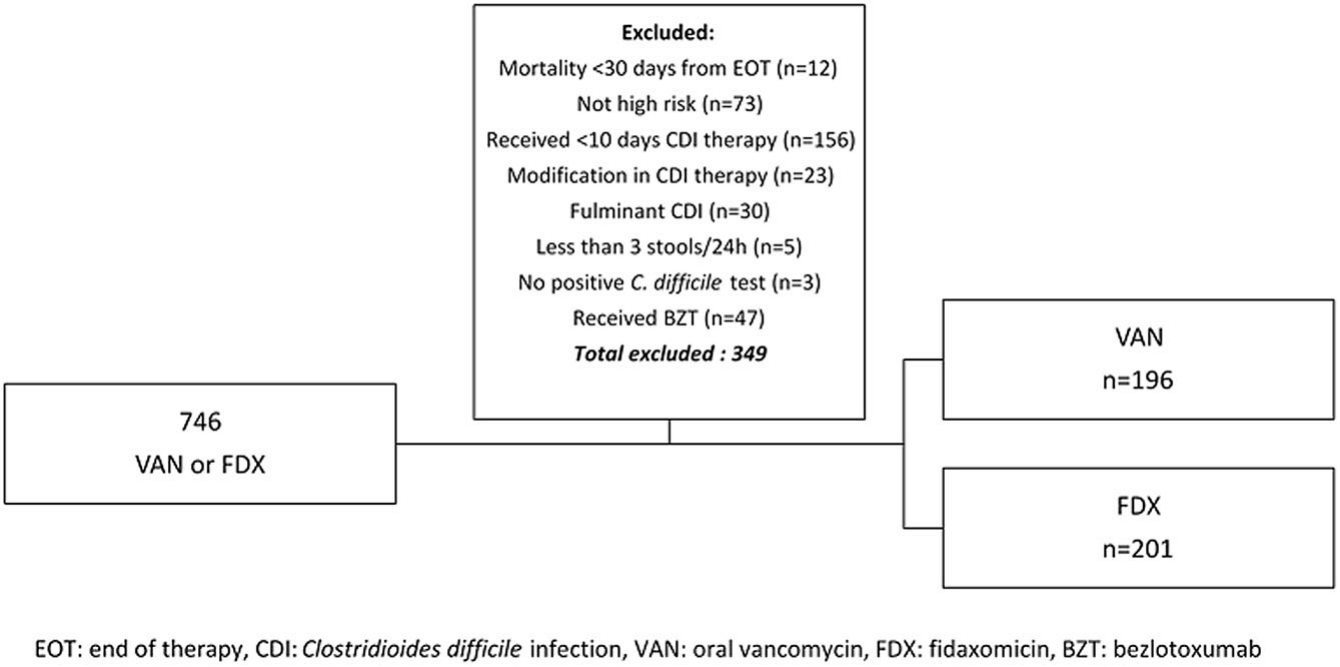
Screening and intervention group distribution.

Of 746 patients who received VAN or FDX during the study period, 397 met inclusion criteria with 196 patients who received VAN and 201 patients who received FDX for presumptive CDI. Reasons for exclusion are shown in Figure 1. Baseline characteristics shown in Table 1 were similar between the two groups. Although there was no difference in the number of immunocompromised patients, there were more patients in the VAN group who had undergone a hematopoietic stem cell transplant (7% vs 2%, *P* = 0.03) and more patients in the FDX group who were receiving immunosuppressive therapy (13% vs 21%, *P* = 0.04).

**Table 1.** Baseline characteristics

	VAN (n = 196)	FDX (n = 201)	*p*-value
Age (mean)	60.6 ± 17.6	63.1 ± 17.5	0.16
Gender (M, (%))	91 (46)	90 (45)	0.74
Race/ethnicity			
Black/African American	128 (65)	126 (63)	0.59
White	59 (30)	57 (28)	0.70
Asian/ Mideast Indian	5 (3)	3 (1)	0.50
More than 1 race	6 (3)	10 (5)	0.44
Hispanic/LatinX	0 (0)	1 (0.5)	1.00
Other	0 (0)	0 (0)	1.00
Unknown	0 (0)	4 (2)	0.12
Immunosuppressive therapy	26 (13)	42 (21)	0.04
Hematologic malignancy	27 (14)	30 (15)	0.74
Solid organ transplant	22 (11)	33 (16)	0.13
Stem cell transplant	14 (7)	5 (2)	0.03
Solid Tumor, chemotherapy within past 3mos	14 (7)	19 (9)	0.40
Inflammatory bowel disease/Crohns disease	11 (6)	9 (4)	0.60
Advanced HIV (CD4 <200 cells/mm^3^)	3 (2)	1 (0.5)	0.37
Number of prior of CDI episodes			
1	28 (14)	31 (15)	0.75
2	9 (5)	8 (4)	0.76
>2	0 (0)	4 (2)	0.12
Risk Factors for rCDI			
Age 65 or older	103 (53)	113 (56)	0.46
Severe CDI	114 (58)	110 (55)	0.49
Immunocompromised	80 (41)	87 (43)	0.62
Potential symptom confounders			
Laxative administration within the last 48h	32 (16)	27 (13)	0.42
Concomitant Antibiotics	31 (16)	21 (10)	0.11
Recent chemotherapy	15 (8)	24 (12)	0.15
GI comorbidity (eg, IBD, diverticulitis, pancreatic insufficiency)	9 (5)	15 (7)	0.23
Mycophenolate	12 (6)	17 (8)	0.37
Tube feeds	17 (9)	12 (6)	0.30
Other infection, associated with diarrhea (eg, COVID, CMV colitis, EPEC, H. pylori)	11 (6)	6 (3)	0.20

There was no difference in rates of presumptive rCDI episodes between those who received VAN versus FDX (9.3% vs 10%, *P* = 0.86) (Table 2). Rates of subsequent CDI episodes within 12 months of completion of therapy and 30-day CDI-related readmissions were also similar (19.4% vs 26%, *P* = 0.12 and 3% vs 4.5%, *P* = 0.6; respectively).

**Table 2.** Outcomes

All Included Patients	VAN (n = 196)	FDX (n = 201)	*p*-value
Recurrence at 8 weeks	18 (9.2)	20 (10)	0.86
Subsequent episode within 12 months	38 (19.4)	53 (26)	0.12
Readmission (30d), CDI-related	6 (3)	9 (4.5)	0.60

There was no difference between groups with respect to the number of confounders that could alternatively explain diarrhea symptoms, including the use of laxatives within the prior 48 hours of starting CDI therapy (VAN 16% vs FDX 13%, *p* = 0.42). An additional subgroup analysis was performed among patients who did not receive laxatives within 48 hours (VAN n = 165, FDX n = 174) which demonstrated similar rates of rCDI (VAN 10% vs FDX 10%, *P* = 0.84). Among this subgroup, there was also no difference in rates of subsequent episodes of CDI in the following 12 months or in rates of 30-day CDI readmissions.

Additional subgroup analyses were performed to assess if any one risk factor of immunocompromised status, age ≥ 65 years, or severe CDI or any combination of these risk factors contributed to differences in outcomes with FDX versus VAN (Table 3). None of the subgroup analyses identified a particular risk factor alone or in combination that resulted in a significant improvement with FDX compared to VAN in the outcomes evaluated. There was a trend toward lower rates of rCDI and subsequent CDI episodes within 12 months in the VAN vs FDX groups among immunocompromised patients, however the difference was not statistically significant.

**Table 3.** Subgroup analyses

Immunocompromised only	VAN (n = 80)	FDX (n = 87)	*p*-value
Recurrence at 8 weeks	11 (14)	8 (9)	0.35
Subsequent episode within 12 months	25 (31)	16 (18)	0.07
Readmission (30d), CDI-related	1 (1)	3 (3)	0.60
Immunocompromised + Age	VAN (n = 20)	FDX (n = 24)	*p*-value
Recurrence at 8 weeks	4 (20)	2 (8)	0.39
Subsequent episode within 12 months	5 (25)	4 (17)	0.71
Readmission (30d), CDI-related	0 (0)	1 (4)	1.00
Immunocompromised + Severe CDI	VAN (n = 15)	FDX (n = 12)	*p*-value
Recurrence at 8 weeks	2 (13)	0(0)	0.49
Subsequent episode within 12 months	5 (33)	1 (8)	0.18
Readmission (30d), CDI-related	0 (0)	0 (0)	1.00
Age only	VAN (n = 35)	FDX (n = 42)	*p*-value
Recurrence at 8 weeks	2 (6)	2 (5)	1.00
Subsequent episode within 12 months	2 (6)	14 (33)	0.004
Readmission (30d), CDI-related	1 (3)	3 (7)	0.62
Severe CDI only	VAN (n = 48)	FDX (n = 37)	p-value
Recurrence at 8 weeks	2 (4)	4 (11)	0.40
Subsequent episode within 12 months	6 (13)	15 (40)	0.19
Readmission (30d), CDI-related	0 (0)	1 (3)	0.43
Severe CDI + Age	VAN (n = 33)	FDX (n = 35)	*p*-value
Recurrence at 8 weeks	3 (9)	6 (17)	0.48
Subsequent episode within 12 months	5 (15)	8 (23)	0.42
Readmission, CDI-related	1 (3)	2 (6)	1.00
MDRO Colonization	VAN (n = 196)	FDX (n = 201)	*p*-value
Vancomycin-resistant *Enterococcus*	1 (0.5)	7 (3.5)	0.07
Extended spectrum ß-lactamase *Enterobacterales*	1 (0.5)	13 (6.5)	0.0016

We also evaluated whether or not being immunocompromised with a history of at least 1 prior episode of presumptive CDI within the past 12 months would result in improved outcomes with FDX (VAN n = 37, FDX n = 43), there also no significant difference in rates of rCDI (VAN 11% vs FDX 5%, *P* = 0.36) or with the other outcomes evaluated.

Overall, 51% of the included patients in this study had a presumptive CDI diagnosis based on a positive stool PCR test (49% of those in the VAN group and 54% in FDX), while the remaining had a positive rectal screen only. In post-hoc analysis to determine if outcomes differed according to sample tested (stool vs rectal screen), there were no differences in outcomes between those receiving VAN versus FDX. There were also no differences in outcomes based on if treatment was started < = 7 days or >7 days from the time of test. The mean time-from-test to initiated therapy was 3.3 days VAN versus 3.6 days FDX.

Rates of VRE and ESBL colonization following CDI treatment were low in both groups. VRE colonization was similar between groups (0.5% VAN vs 3.5% FDX, *P* = 0.07) and more patients receiving FDX had ESBL colonization (0.5% vs 6.5%, P< 0.01).

## Discussion

Among patients at risk for rCDI, the use of FDX was not associated with reduced rates of presumptive rCDI episodes, subsequent episodes of presumptive CDI within 12 months, or 30-day CDI-related readmissions compared to VAN. Additional subgroup analyses did not identify any one particular risk factor for recurrence, alone or in combination, to be associated with improved outcomes with FDX over VAN with respect to the outcomes evaluated in this study.

Although FDX has been shown in clinical studies to be associated with lower rates of recurrence (by 5.8%–14.2%), the data is primarily derived from large clinical trials that included patients regardless of their baseline risk for recurrence.^2–5^ Some studies have sought to determine whether the benefits of FDX on lower rates of recurrence remains among patients that may be at greater risk for recurrence and the data are somewhat conflicting. Additional subgroup analyses of the EXTEND trial, comparing the use of FDX using an extended dosing regimen to VAN among patients 60 years of age and older evaluated rates of rCDI in addition to clinical cure.^7^ This study evaluated patients by advanced age (≥75 years old vs 60–74 years), cancer diagnosis, CDI severity, prior CDI occurrences and infections caused by the hypervirulent NAP1/B1/027 strain. In the rCDI analysis, there was no between-treatment difference in the incidence of recurrence among the subgroups included. Conversely, subgroup analyses from two other double-blind randomized controlled trials assessing outcomes among 183 patients with cancer found lower rates of recurrence among those receiving FDX over VAN (13.5% vs 29.6%, *P* = 0.018).^8^ Another study evaluated a composite outcome of clinical failure, relapse at 30 days, or CDI-related death in a cohort of immunocompromised patients (including solid organ and hematopoietic stem cell transplant patients, those undergoing induction chemotherapy, and those receiving immunomodulatory agents).^9^ Two-hundred and thirty-eight patients were included in this study (38 patients received FDX and 200 VAN), 4 (10.5%) in the FDX group versus 38 (19%) met the composite outcome. After adjusting for confounding variables on multivariate analysis, FDX was associated with a 72% reduction in meeting the composite outcome compared to those that received VAN (hazard ratio [HR], 0.28; 95% CI 0.08–0.93).^9^ A recent meta-analysis identified five studies including 3 randomized controlled trials and 2 observational studies where rates of rCDI with FDX versus VAN were assessed based on CDI severity. Among patients with severe CDI, rates of recurrence were found to be 40% lower with FDX compared to VAN (19% vs 25.7% ; RR 0.6, 95%CI 0.37–0.97).^10^ An open-label randomized trial comparing FDX and VAN among hospitalized patients receiving concomitant antibiotics, found no significant difference in rates of clinical cure (73% FDX vs 63% VAN, *P* = 0.195) or rCDI (3.3% FDX vs 4% VAN, *P* > 0.99).^11^ As the incidence of CDI continues to rise with a disproportionate rise in rCDI, potentially relating to increased abundance of hypervirulent strains, understanding the best treatment for rCDI is of paramount importance.^12^ Our study is representative of real-world data including patients with target risk factors for recurrence (age > = 65 yr, immunocompromised status, and/or severe CDI) for which we based a clinical pathway on. The results of our study offers insight into whether FDX is associated with lower rates of recurrence among these higher risk groups outside the context of a clinical trial.

Consensus guidelines can be of great utility in forming the framework for developing institution-specific treatment pathways for managing various infectious diseases and establishing evidence-based standard of care. The 2021 IDSA/SHEA guidelines for the management of CDI primarily based recommendations on the findings of 4 clinical trials that they included in a pooled analysis.^1^ In their analysis, the combined rates of sustained clinical response at 4 weeks was significantly increased among those receiving FDX compared to VAN. However, sustained clinical response at 90 days and mortality were no different between groups. Despite the lack of observed benefit in sustained clinical cure beyond 30 days or mortality, the guidelines recommend FDX as first-line therapy. The rationale outlined in the IDSA/SHEA guidance for FDX as prefered therapy include: (1) high value is placed on avoiding rCDI based on quality of life indicators, (2) use of FDX, despite the high acquisition cost, is cost-effective based on cost-effectiveness analyses, (3) FDX may be more feasible with regard to less frequent dosing, (4) is acceptable to patients and their providers. Following the availability of the 2021 IDSA/SHEA update to the CDI management guidelines recommending with the moderate certainty evidence the preference for FDX based on the pooled analyses, we opted to change our institutional pathway to recommend FDX as first-line therapy. However, due the cost of therapy and known challenges with insurance coverage we added the requirement that patients have at least one risk factor for CDI recurrence in an attempt to target patients most likely to benefit. With this change, our medical center experienced a significant increase in expenditures on FDX and our utilization compared to other similar medical centers based on benchmarking data was an evident outlier. We also invest a large amount of clinician, clinical pharmacist, and antimicrobial stewardship workforce time in reviewing patients with CDI by ensuring appropriate treatment selection and facilitating outpatient coverage of FDX for patients who may be discharged prior to completion of their CDI therapy. Given these factors, it was pertinent to complete this review of outcomes data to ensure that this practice is resulting in meaningful improvements in clinical outcomes for our patients. With the findings of this study, the preference for FDX, at least in patients meeting our high risk for rCDI criteria, is not seemingly justified. As a result, we intend to make changes to our institutional CDI treatment pathway.

One potential clinical advantage to using FDX is its narrower spectrum of activity resulting in less disruption of the gut microbiota which in turn may result in lower rates of multidrug-resistant organism colonization. Prior studies have found FDX to have less deleterious effects on the intestinal microbiota and subsequently associated with lower susceptibility to VRE and ESBL colonization compared to VAN.^13,14^ We however did not observe this advantage of FDX over VAN. In our analysis, the rate of ESBL colonization was significantly higher with FDX vs VAN (6.5% vs 0.5%, *P* < 0.01) and there was no difference in rates of VRE. Overall the rates of VRE and ESBL colonization in both groups were quite low (3.5% FDX, 0.5% VAN, *P* = 0.07) making it difficult to make definitive conclusions regarding risk for colonization between agents. There are also a number of confounding variables that might influence this outcome that make this data difficult to interpret.

There are a number of limitations to our study. Given the method in which included patients were diagnosed as having CDI, based on an initial screen and initiation of treatment when signs and symptoms consistent with CDI developed, we were only able to report on outcomes among patients with a presumptive CDI diagnosis. The results of our study, therefore, may not be applicable to other hospitals where a diagnosis is made using a different approach. We did however assess for the presence of other confounding factors that could have contributed to diarrheal symptoms by performing subgroup analyses that removed patients with some of these confounders. We also performed subgroup analyses to assess if diagnosis using rectal screen sample versus stool sample testing had an impact on outcomes. In these subgroup analyses, we did not observe a clear benefit of FDX over VAN. Due to the retrospective nature of this study, we relied on patient charts to assess baseline characteristics, presence of risk factors, and outcomes for which there is a chance that information was lacking accuracy or an insufficient amount of detail was available to fully assess certain data points (eg, number of stools to confirm CDI diagnosis). We also could have missed recurrences of infection, subsequent CDI infections, and readmissions for patients that may have sought care elsewhere after their index infection. Over the course of the study, multiple distinct waves of COVID-19 occurred, and rates CDI hospitalization gradually decreased nationwide.^15^ It is possible these epidemiologic trends affected the results of this study. Additionally, our CDI diagnostic algorithm and use of NAAT may result in the over diagnosis of CDI. Approximately 15% of patients received laxatives within 48h of starting CDI therapy, which could have resulted in unnecessary treatment and could have skewed the observed outcomes. However as noted previously, a subgroup analysis was performed to remove patients who received laxatives, and there remained no difference in rates of recurrence or the other outcomes evaluated. Lastly, we may not have had a large enough sample size to identify statistically significant differences in outcomes for the primary and subgroup analysis.

## Conclusion

Targeting the use of FDX for CDI to patients at high risk for recurrence did not improve the primary outcome (rates of presumptive rCDI), or secondary outcomes (subsequent CDI episodes within 12 months, or CDI-related 30-day readmissions) compared to VAN among patients with a presumptive CDI diagnosis. We observed a trend toward improved outcomes with FDX over VAN among immunocompromised patients, however a larger sample size is needed to more accurately assess outcomes in this patient population. Further investigation is warranted to establish patient populations that would benefit the most from FDX.
